# The effects of an ecdysteroid supplement on indices of physical and mental performance

**DOI:** 10.1080/15502783.2025.2550140

**Published:** 2025-08-25

**Authors:** Antonio Crisanti, Tobin Silver, Lia Jiannine, Jose Antonio

**Affiliations:** Nova Southeastern University, Exercise and Sport Science, Davie, FL, USA

**Keywords:** Dietary supplement, mood, sleep, strength

## Abstract

**Background:**

Turkesterone is a naturally occurring ecdysteroid found in certain plants and insects. With a chemical structure like testosterone, it has been purported to have an anabolic effect despite the lack of human data. Thus, this investigation aimed to determine whether four weeks of supplementing with a turkesterone-containing product affected body composition, handgrip strength, mood, and sleep quality in healthy, young, university male and female participants.

**Materials and methods:**

Twenty-four participants (12 male and 12 female; mean ± SD: age 22 ± 5; height 170.4 ± 8.6 cm; body mass 71.3 ± 20.1 kg) volunteered for this investigation. Participants were randomly assigned to a treatment or placebo group in a double-blind fashion. They were pre-tested for body composition (Inbody270), handgrip strength, Profile of Mood States (POMS), and sleep quality (Pittsburgh Sleep Quality Index or PSQI). Then, they supplemented daily for four weeks with either the treatment (i.e. one capsule daily of Turk Builder®) or placebo (i.e. one capsule daily of cellulose). Participants were instructed not to change their dietary or exercise habits. An unpaired t-test was used to compare the delta scores of the treatment vs. placebo.

**Results:**

There were no significant pretest differences for any of the measures between the groups. Moreover, there were no significant differences in the delta score between groups for any of the assessments (i.e. body composition, handgrip, POMS, and PSQI).

All data are expressed as the mean ± standard deviation. Legend: BM – body mass, FFM – fat-free mass, FM – fat mass, HG – handgrip, PSQI – Pittsburgh Sleep Quality Index, TBW – total body water, TMDS – total mood disturbance score. There were no significant differences between groups for the delta score.

**Conclusions:**

Based on a limited sample size as well as short treatment duration, there is no evidence that this over-the-counter dietary supplement affects body composition, handgrip strength, POMS, or PSQI.

**Table 1.** Physical and mental assessments.

	Treatment			Placebo			
	Pre	Post	Delta	Pre	Post	Delta	p-value delta
BM kg	68.0 ± 13.1	67.8 ± 12.6	−0.2 ± 1.7	74.6 ± 20.1	75.7 ± 21.6	1.1 ± 1.8	0.0825
FFM kg	52.9 ± 11.5	52.7 ± 10.9	−0.2 ± 1.5	58.2 ± 17.2	59.6 ± 19.0	1.4 ± 2.7	0.1064
FM kg	15.0 ± 5.6	15.1 ± 5.3	0.1 ± 1.2	16.4 ± 8.1	16.2 ± 7.9	−0.3 ± 1.6	0.5529
% Fat	22.3 ± 7.2	22.4 ± 6.8	0.2 ± 1.6	22.1 ± 9.7	21.7 ± 9.8	−0.3 ± 1.9	0.4912
TBW liters	38.7 ± 8.4	38.6 ± 8.0	−0.1 ± 1.1	42.6 ± 12.6	43.6 ± 13.9	1.0 ± 1.0	0.1088
HG kg	43 ± 12	46 ± 10	2 ± 9	50 ± 16	47 ± 17	−3 ± 6	0.2450
TMDS	14.1 ± 21.8	10.8 ± 16.6	−3.3 ± 15.4	4.7 ± 13.4	5.3 ± 16.2	0.6 ± 16.1	0.5577
PSQI	7.9 ± 2.6	7.0 ± 2.9	−0.9 ± 2.0	7.1 ± 3.4	7.3 ± 4.0	0.3 ± 1.8	0.1705

